# Rapid Genome Sequencing of RNA Viruses

**DOI:** 10.3201/eid1302.061032

**Published:** 2007-02

**Authors:** Tetsuya Mizutani, Daiji Endoh, Michiko Okamoto, Kazuya Shirato, Hiroyuki Shimizu, Minetaro Arita, Shuetsu Fukushi, Masayuki Saijo, Kouji, Sakai, Chang Kweng Lim, Mikako Ito, Reiko Nerome, Tomohiko Takasaki, Koji Ishii, Tetsuro Suzuki, Ichiro Kurane, Shigeru Morikawa, Hidekazu Nishimura

**Affiliations:** * National Institute of Infectious Diseases, Tokyo, Japan; † Rakuno Gakuen University, Ebetsu, Japan; ‡ Sendai Medical Center, Sendai, Japan

**Keywords:** Emerging infectious virus, SARS-CoV, West Nile virus, rapid determination system, whole genome amplification, direct sequencing, dispatch

## Abstract

We developed a system for rapid determination of viral RNA sequences whereby genomic sequence is obtained from cultured virus isolates without subcloning into plasmid vectors. This method affords new opportunities to address the challenges of unknown or untypeable emerging viruses.

Over the past few years, global migration has led to emerging infectious diseases that pose substantial risks to public health. To prevent potential outbreaks, early detection of infectious pathogens is necessary. In particular, the recent outbreak of severe acute respiratory syndrome (SARS) provided important lessons on how unknown viruses should be detected rapidly. Thus, a standardized and qualified system is required for rapid nucleic acid sequence determination for newly emerging viruses.

Recently, we developed a new method for detecting RNA viruses. This method, based on cDNA representational difference analysis (cDNA RDA), uses 96 hexanucleotides that are not suitable for priming ribosomal RNAs but that normally prime most of the genome of an RNA virus as primers for reverse transcription in cDNA RDA (*1*). However, the RDA method with a cloning step requires at least 1 week for the determination of the nucleic acid sequence.

## The Method

**Figure 1:**
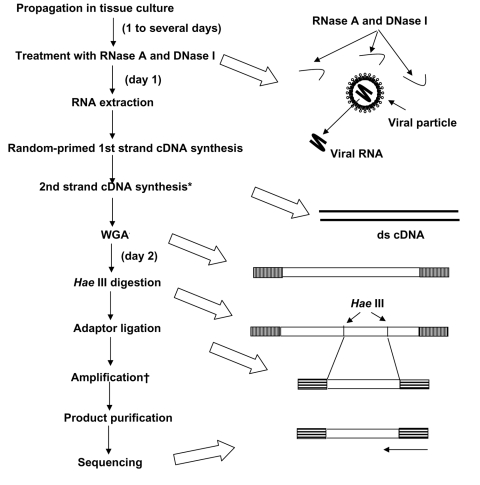
Overall scheme of the rapid determination of viral RNA sequence method. *By adding RNase H; WGA, whole genome amplification; †With specially designed primer sets as shown in Figure 2.

Our new system for rapid determination of viral RNA sequence (RDV) uses whole-genome amplification and direct sequencing techniques (Figure 1). The RDV method comprises 6 procedures: 1) effective destruction of cellular RNA and DNA for semipurification of viral particles, 2) effective elimination of DNA fragments by using a prefiltration column system and elution of small amounts of RNA, 3) effective synthesis of first- and second-strand cDNAs, 4) construction and amplification of a cDNA library, 5) construction of a second cDNA library, and 6) direct sequencing using optimized primers. The RDV method enables a broad range of partial nucleotide sequences within the entire viral RNA genome to be obtained within 2 days without cloning into plasmids.

To eliminate contaminating cellular RNA and DNA from the samples, 0.001 μg of RNase A (Qiagen, Hilden, Germany) and 1 μL (2 U) of Turbo DNA-free DNase I (Ambion, Austin, TX, USA) with 1× Turbo DNA-free buffer were incubated at 37°C for 30 min under conditions that prevented destruction of viral RNA in the viral particles. The RNA in the viral particles was then extracted within 30 min by using a total RNA isolation mini kit (Agilent Technology Inc., Palo Alto, CA, USA). We confirmed that DNA was effectively eliminated by this RNA extraction kit.

In accordance with the Invitrogen manual, cDNA was synthesized, by using random hexamers (Takara Bio Inc., Kyoto, Japan) and Superscript III (Invitrogen, Carlsbad, CA, USA) lacking RNase H activity, at 50°C for 1 h. Then 60 U of RNase H (Takara Bio Inc.) added before synthesis of second-strand cDNA at 50°C for 1 h. In accordance with the manual, a whole genome amplification system (WGA; Sigma-Aldrich, Saint Louis, MO, USA), which was developed for amplification of genomic DNA, was used to amplify viral double-stranded cDNA. This process was performed within 90 min. Instead of the Taq polymerase recommended in the kit, we used 1.25 U of AmpliTaq Gold LD (Applied Biosystems, Foster City, CA, USA) to obtain a high yield of the PCR products. Primers were provided in the WGA kit, but no information regarding their sequences was obtained. The reaction mixture was heated at 95°C for 9 min (for activation of AmpliTaq Gold), followed by 70 cycles of amplification using Mastercycler (Eppendorf AG, Hamburg, Germany). Each PCR cycle consisted of annealing at 68°C for 1 min, primer extension at 72°C for 5 min, and denaturation at 94°C for 1 min.

**Figure 2:**
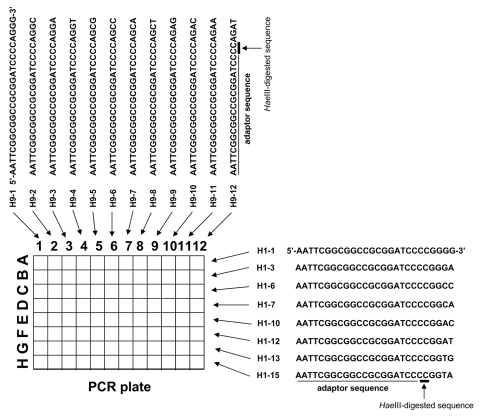
Primers used in rapid determination of viral RNA sequence method.

The 1st cDNA library was digested with 40 U of *Hae*III (Takara Bio Inc.) at 37°C for 30 min. DNA was purified by using the MonoFas DNA isolation system (GL Science, Tokyo, Japan), and a blunt *EcoR*I-*Not*I-*Bam*HI adaptor (10 pmol; Takara Bio Inc.) was ligated at 16°C for 30 min by using DNA Ligation Kit, Mighty Mix (Takara Bio Inc.). The second cDNA library was amplified by PCR with specially designed primer sets in which 6 nucleotides composed of CC (*Hae*III-digested sequence) and 4 variable nucleotides were added to the 3′ end of the adaptor sequence (Figure 2). For example, 1 primer set was as follows: forward primer, H1–1: 5′-AATTCGGCGGCCGCGGATCC*CC*GGGG-3′; reverse primer H9–3: 5′-AATTCGGCGGCCGCGGATCC*CC*AGGA-3′ (the adaptor sequence is underlined, and the *Hae*III-digested sequence is shown in italics) (Figure 2).

We always used >12 primer sets and 0.83 μmol of each primer per cDNA library. PCR was performed with AmpliTaq Gold Master Mix (Applied Biosystems). The reaction mixture was heated at 95°C for 12 min, followed by 70 cycles of amplification. Each PCR cycle consisted of annealing and primer extension at 72°C for 30 s and denaturation at 94°C for 30 s. A single band was consistently obtained in ≈50% of the reactions. DNA was purified from the PCR by using MonoFas. Occasionally, we purified DNA fragments from the gels when >2 bands were detected. Direct sequencing was performed with the forward primer, reverse primer, or both.

When the number of viral particles in the sample was high, we omitted the RNase A and DNase I treatments and used the RNeasy Mini Kit (Qiagen) for RNA extraction. We occasionally used a whole transcriptome amplification kit (Rubicon Genomics Inc, Ann Arbor, MI, USA) instead of the WGA kit because both kits yielded similar amplification results.

In preliminary studies that used referential RNA viruses, we attempted to determine the nucleic acid sequences of SARS coronavirus, mouse hepatitis virus, West Nile virus, Japanese encephalitis virus, and dengue virus type 2 in culture supernatants (10–100 μL) by using the RDV method. The percentages of positive fragments (number of fragments containing viral nucleic acid/total number of sequenced fragments) in the reactions for detection of these 5 viruses were 60% (3/5), 45% (5/11), 100% (12/12), 50% (5/10), and 40% (4/10), respectively. As a clinical application, a throat swab specimen from a patient with fever and upper respiratory infection was characterized. Although the specimen exhibited enterovirus-like cytopathic effect by inoculation into HEF and GMK cells when cell culture system for virus isolation was used (*2*), extracted RNA from the supernatant of the cells showed no amplification by reverse transcription–PCR (RT-PCR) when 1 of the conventional primer sets for human enteroviruses was used (*3*,*4*). In the cell culture supernatant analysis by the RDV method, the specimen exhibited amplification of the partial nucleotide sequences of coxsackie A14 virus (nucleotide sequence data are available in the DDBJ/EMBL/GenBank databases under accession nos. AB275848–AB275853). Thus, the RDV method could detect unidentified cytopathic-effect agents such as enterovirus that could not be detected by RT-PCR when the conventional primer set for enteroviruses was used.

## Conclusions

The RDV method is a rapid method for the direct determination of viral RNA sequences without using the cDNA cloning procedure. The limitations of the RDV method are the requirement for cell culture isolate and the large number of steps. However, RDV would be useful for species-independent detection of RNA viruses including unknown or untypeable emerging RNA viruses. Furthermore, with minor modifications, this method would also be applicable to the detection of DNA viruses and bacteria.
